# Structures of *Medicago truncatula* L-Histidinol Dehydrogenase Show Rearrangements Required for NAD^+^ Binding and the Cofactor Positioned to Accept a Hydride

**DOI:** 10.1038/s41598-017-10859-0

**Published:** 2017-09-05

**Authors:** Milosz Ruszkowski, Zbigniew Dauter

**Affiliations:** Synchrotron Radiation Research Section of MCL, National Cancer Institute, Argonne, IL USA

## Abstract

Plants, lower eukaryotes, bacteria, and archaebacteria synthesise L-histidine (His) in a similar, multistep pathway that is absent in mammals. This makes the His biosynthetic route a promising target for herbicides, antifungal agents, and antibiotics. The last enzyme of the pathway, bifunctional L-histidinol dehydrogenase (HDH, EC 1.1.1.23), catalyses two oxidation reactions: from L-histidinol (HOL) to L-histidinaldehyde and from L-histidinaldehyde to His. Over the course of the reaction, HDH utilises two molecules of NAD^+^ as the hydride acceptor. The object of this study was the HDH enzyme from the model legume plant, *Medicago truncatula* (*Mt*HDH). Three crystal structures complexed with imidazole, HOL, and His with NAD^+^ provided in-depth insights into the enzyme architecture, its active site, and the cofactor binding mode. The overall structure of *Mt*HDH is similar to the two bacterial orthologues whose three-dimensional structures have been determined. The three snapshots, with the *Mt*HDH enzyme captured in different states, visualise structural rearrangements that allow for NAD^+^ binding for the first time. Furthermore, the *Mt*HDH complex with His and NAD^+^ displays the cofactor molecule situated in a way that would allow for a hydride transfer.

## Introduction

L-Histidine (His) biosynthesis is a part of the primary anabolism of archaebacteria, bacteria, lower eukaryotes, and plants. In a multistep pathway, His is synthesised from 5-phosphoribosyl-1-pyrophosphate and ATP, which links amino acid and nucleotide metabolism^1, 2^. The absence of the His-biosynthetic pathway in mammals makes it a good target for antibiotics, herbicides, and antifungal agents^3–5^. In multiple examples, the His-auxotrophic mutants, lacking one or more of the His-synthesising enzymes, are unable to tolerate His starvation. For instance, in *Mycobacterium tuberculosis* and *Brucella suis*, His-synthesising enzymes are essential for survival of those bacterial species, responsible for very severe infections, tuberculosis and brucellosis, respectively^6, 7^. Among all enzymes from the His-biosynthetic pathway, the ultimate catalyst, L-histidinol dehydrogenase (HDH), deserves special attention^8^. Blocking the last step of a biosynthetic pathway not only hinders the final product but also results in the accumulation of intermediate metabolites, which may increase the toxic effect resulting from the lack of the pathway end-product alone. In fact, HDH has been prioritised among the top 50 targets against totally drug-resistant strains of *Mycobacterium tuberculosis* ^9^.

**Figure 1:**
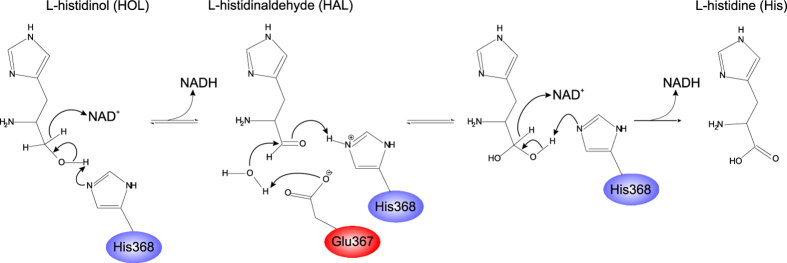
Mechanism of the reaction catalysed by *Mt*HDH. The figure is modified from^12, 16, 39^.

HDH (EC 1.1.1.23) is a bifunctional enzyme that oxidises L-histidinol (HOL) via L-histidinaldehyde (HAL) into His in two sequential reactions, each coupled with a reduction of one nicotinamide adenine dinucleotide (NAD^+^) molecule to NADH (Fig. 1)^10^. The enzyme operates via the Bi-Uni-Uni-Bi Ping Pong mechanism, and, rather than leaving the catalytic site, the intermediate HAL is immediately converted to His^10–13^. It is also known that NAD^+^ binding is effective only to HOL- or HAL-complexed HDHs^11, 12^, which is uncommon for NAD-dependent dehydrogenases. In most cases, the cofactor binds first. HDHs are metalloenzymes, binding a single Zn^2+^ cation per subunit^14, 15^. However, Zn^2+^ is not directly involved in catalysis. Instead, it plays a structural role by facilitating and stabilising the binding of HOL, HAL, and His^16^.

Structural information is crucial for a rationale-based drug design. To date, there have been only two publications presenting crystal structures of HDH enzymes, both from bacterial sources. The enzyme from *E. coli* (*Ec*HisD) (in this work, we use the HisD acronym for bacterial HDHs, which relates to *his* operon organisation in many prokaryotic species) was studied by Barbosa *et al*.^16^. The authors presented four structures: (i) unliganded *Ec*HisD (Protein Data Bank, PDB ID: 1k75) and complexes with (ii) Zn^2+^, HOL, and NAD^+^ (PDB ID: 1kae), and (iii) Zn^2+^ and L-histamine (1kar) and (iv) Zn^2+^ and His (1kah). This set of structures revealed, for the first time, both the overall fold of an HDH enzyme and the location and composition of the active site. Unfortunately, as the authors acknowledged, the enzyme in the structure with HOL and NAD^+^ had been trapped in a state that would not allow the hydride transfer to occur, as the HOL carbon that is to be oxidised and the C4 atom of NAD^+^ are too distant (approximately 4.9 Å from each other). It is worth noting that the distance between the hydride donor and the acceptor should be approximately 3 Å and has been calculated to be 2.7 Å for the transition state^17^. Because the distance in the *Ec*HisD complex was too long, the substrate (HOL) was found in the active site instead of the product (His). The authors attributed this situation to the crystal packing, which prevented small rearrangements that would otherwise allow NAD^+^ to approach and oxidise HOL. In other words, soaking *Ec*HisD crystals with HOL and NAD^+^ did not drive the necessary conformational changes, as the protein chain had been locked by the structure of the crystal lattice^16^.

Two other structures of HDH, from *B. suis* (*Bs*HisD), show the enzyme unliganded (PDB ID: 4g07) and with a very potent inhibitor, (3 *S*)-3-amino-4-(1*H*-imidazol-5-yl)-1-[4-(phenylmethoxy)phenyl]-butan-2-one (4g09)^18^. Two other reports that deal with HDH structures are based on computational modelling^19, 20^. In both cases, as the template for molecular modelling, the *Ec*HisD complex with Zn^2+^, HOL, and NAD^+^ [PDB ID: 1kae, ref. 16] was used in the locked state. Interestingly, the compounds that exhibited the highest inhibition of HDH were predicted to simultaneously occupy the substrate binding site as well as a part of the NAD^+^ binding cleft^19^.

Since bacterial HDHs are perceived as very promising targets for antibiotics, a parallel approach seems reasonable for seeking novel herbicides. Studies of plant His-biosynthetic pathways not only expand the knowledge about this fragment of plant metabolism but are also needed to deduce the differences and similarities between the prokaryotic and eukaryotic routes.

Overall, the plant pathway leading to His biosynthesis is very similar to the bacterial pathway^21, 22^. Most of the enzymes that take part in plant His biosynthesis are encoded by single genes, unlike for many other plant metabolic pathways^21^. All enzymes required for His biosynthesis in plants are located in chloroplasts and have been identified: HisN1^23^; HisN2^24^, HisN3^25^, HisN4^26^, HisN5^23^, HisN6^27^, HisN7^23, 28^, and HisN8^29–31^. However, structural knowledge of plant His-synthesising enzymes is very scarce, with only the structures of *Arabidopsis thaliana* HisN5^32, 33^ and *Medicago truncatula* HisN7^34^ reported to date.

This article is dedicated to structural studies of HisN8, a HDH from the model legume plant *M. truncatula*, which from now on will be referred to as *Mt*HDH. This is the first report of an HDH structure from a plant source. We show three crystal structures of Zn^2+^-bound *Mt*HDH complexed with: (i) imidazole (IMD); (ii) HOL; and (iii) His with NAD^+^. These structures, representing snapshots over the course of the reaction, expand the knowledge of HDH enzymes by showing the movement of the loops triggered by HOL binding as well as revealing the location of NAD^+^ with C4, the hydride-accepting atom within a reacting range to the HOL carbon that is oxidised. Additionally, because HDH enzymes from plants and bacteria are similar, many of the results presented herein should concern prokaryotic HDHs in addition to the orthologs of plant origin. It is important to note that our structures represent the active state of the enzyme, which may serve as an updated model for designing HDH-targeted herbicides and antibiotics.

## Results and Discussion

### General properties of the *Mt*HDH structure

UniProt^35^ search revealed two HDH enzymes in the *M. truncatula* proteome [B7FNC7 and G7IKX3]. The only difference between the two sequences is an Asp79Asn mutation that lies outside the PCR primer-binding region of the *Mt*HDH open reading frame (ORF). DNA sequencing verified that the amplified ORF used in this study corresponds to the UniProt G7IKX3 entry without the predicted chloroplast-targeting signal peptide.

**Figure 2:**
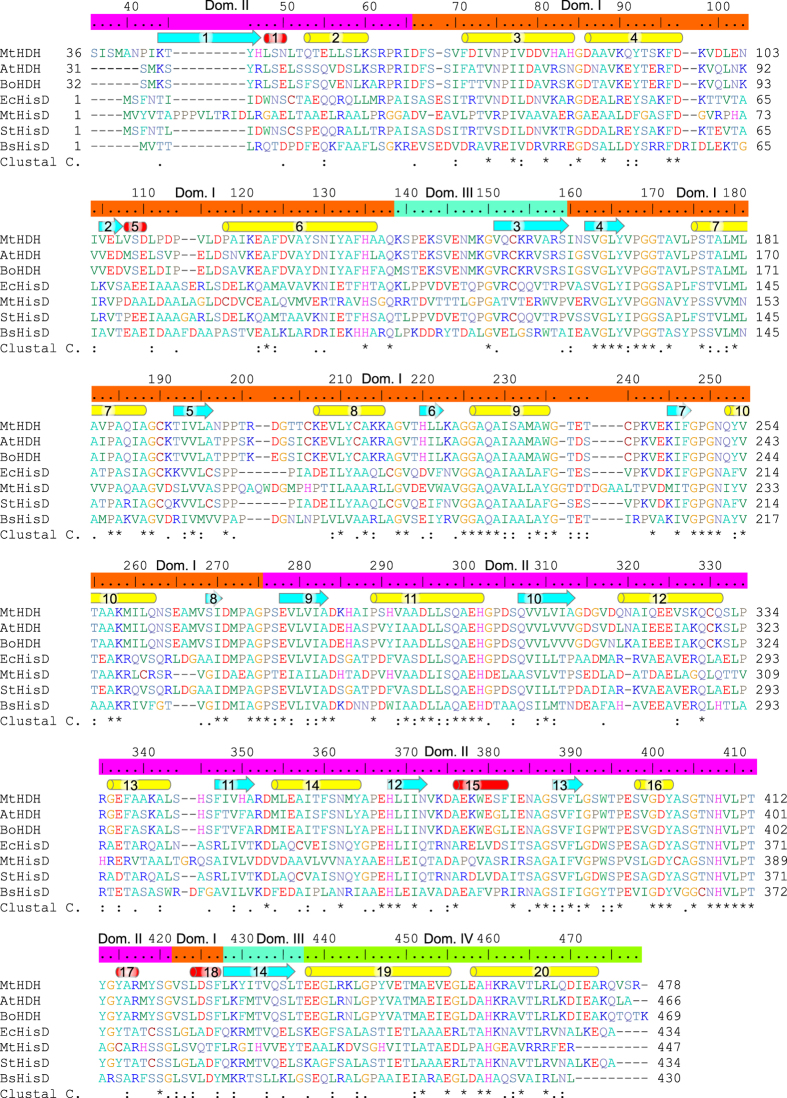
Sequence alignment of HDH enzymes from various sources: *Mt*HDH, *Medicago truncatula* [UniProt accession number: G7IKX3]; *At*HDH, *Arabidopsis thaliana* [Q9C5U8-1]; *Bo*HDH, *Brassica oleracea* [P24226]; *Ec*HisD, *Escherichia coli* (strain K12) [P06988]; *Mt*HisD, *Mycobacterium tuberculosis* (strain H37Rv) [P9WNW9]; *St*HisD, *Salmonella typhimurium* (strain LT2) [P10370]; *Bs*HisD, *Brucella suis* biovar 1 (strain 1330) [Q8G2R2]. For clarity of the alignment, the N-terminal signal peptides of *At* and *Bo* enzymes were truncated at positions 31 and 32, respectively, which correspond to Pro42 in *Mt* structure, which is the first residue visible in the electron density. Domains of MtHDH are coloured: I, orange; II, magenta; III, turquoise; IV, chartreuse. Elements of the secondary structure are: α-helices, yellow; 3_10_ helices, red; β-strands, cyan. Helices are numbered consecutively, regardless of the type.

The sequences of plant HDHs are highly homologous. Even between the plant and bacteria kingdoms, the similarities are significant (Fig. 2). For instance, identities/similarities between the *Medicago* enzyme and orthologs from *A. thaliana*, *B. oleracea*, and *E. coli* are 79%/88%, 78%/87%, and 46%/59%, respectively.

**Figure 3:**
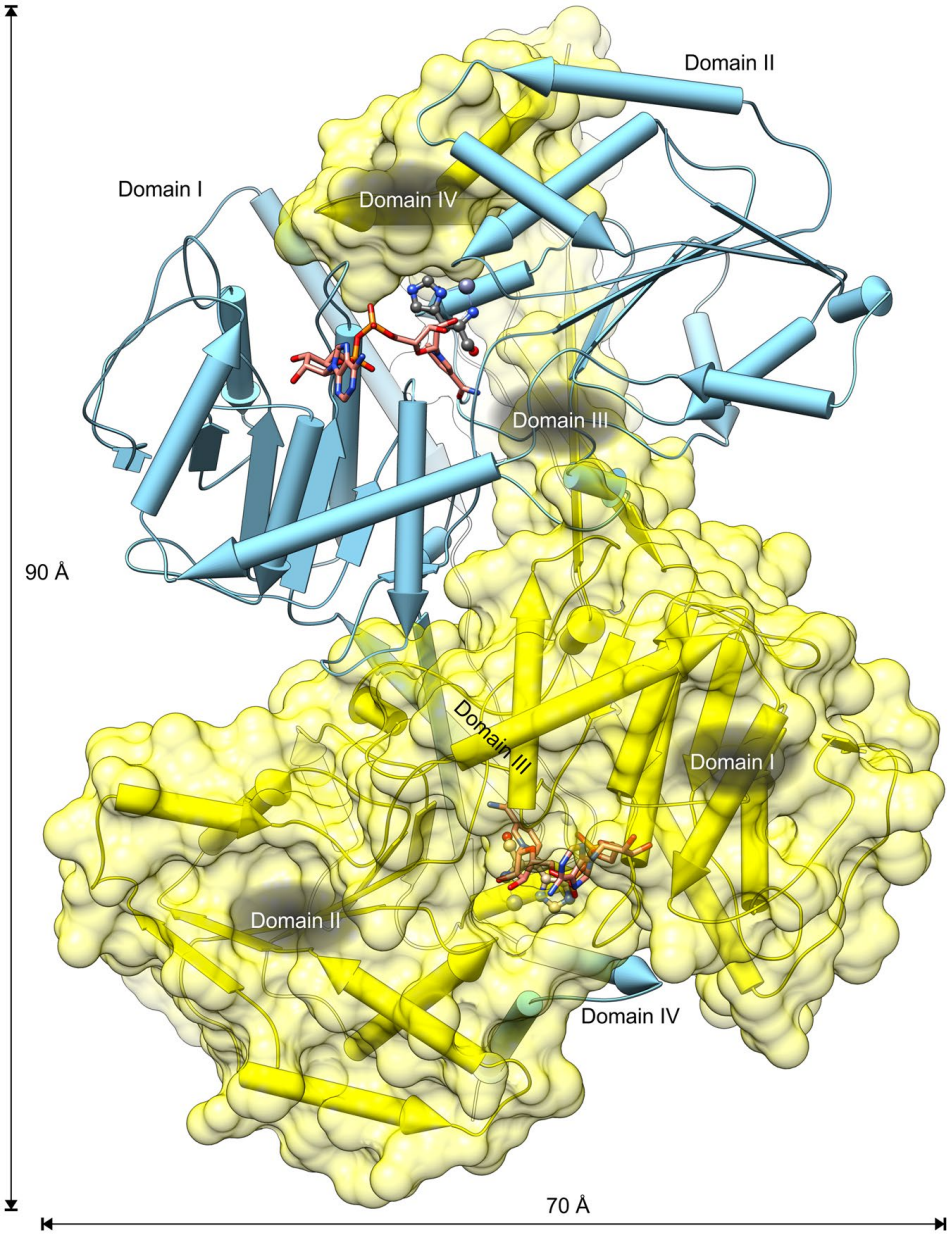
Structure of the *Mt*HDH homodimer. Chain A, cyan, in pipes-and-planks representation and chain B, yellow, with the protein surface are shown. NAD^+^ and L-histidinol (HOL) from superposed structures show locations of the active sites, one per monomer. White-on-black denotation of domains corresponds to the subunit B.

*Mt*HDH crystallises in the *P*2_1_ space group. The three structures reported herein are isomorphous, with three dimers (AB, CD, and EF) forming the asymmetric unit. The dimeric quaternary structure agrees with the results of size-exclusion chromatography (not shown) as well as the oligomeric state reported for other HDH enzymes^16, 18^. The *Mt*HDH homodimeric assembly (Fig. 3) measures approximately 90 × 70 × 50 Å. Based on *PISA* ^36^ calculations, the inter-subunit interface is above 5,200 Å^2^ (per monomer), which is approximately 25% of the total solvent-accessible area. Along with numerous hydrophobic contacts, 25 salt bridges and at least 90 hydrogen bonds stabilise a *Mt*HDH dimer. For clarity, the following structural analyses are based on the A subunits (of the three structures), unless noted otherwise.

The *Mt*HDH monomer is composed of four domains. Domain I (residues 66–138, 160–275, and 422–427) contains a core with a Rossmann-fold-like super-secondary structure^37^. The six-stranded β-sheet (ordered 2–6–5–4–7–8), which lies in the centre of domain I, is mostly parallel except for the antiparallel strand 2 at the edge of the β-sheet. The β-sheet is sandwiched between helices α7 and α8 from one side and α9 and α10 from the other. The Rossmann-fold core of domain I is surrounded by helix α6, which is interacting with α7 and α8, and by a V-shaped pair, α3 and α4, that shields α9 and α10. Additionally, a short 3_10_ helix η18 is formed at the interface between domains I and III.

Domain II (residues 36–65 and 276–421), also adopts the Rossmann-like fold in its core, despite a very different sequence. The six-stranded, parallel β-sheet (ordered 1–11–10–9–12–13) is surrounded by helices α11, α12, α13, and α16 on one side and η1, α2, α14, η15, and η17 on the other. Domains I and II together form an extended globular structure with a cleft at the interface.

In the *Mt*HDH dimer, the β-sheet of domain II is extended from the edge of the β13 strand by two strands belonging to domain III (residues 139–159 and 428–437): β14 (parallel) and β3 (anti-parallel) of the second protein subunit. Domains III and IV (residues 438–478) are almost perpendicular to each other. They form an L-shaped structure that is mutually swapped between the subunits and occupies a cleft between domains I and II of the dimer-mate subunit. The C-terminal domain IV (helices α19 and α20) is V-shaped and interacts mostly with domain II of the other subunit, completing the active site of its dimer-mate (see below).

### Complex with imidazole illustrates a state before the reaction

**Figure 4:**
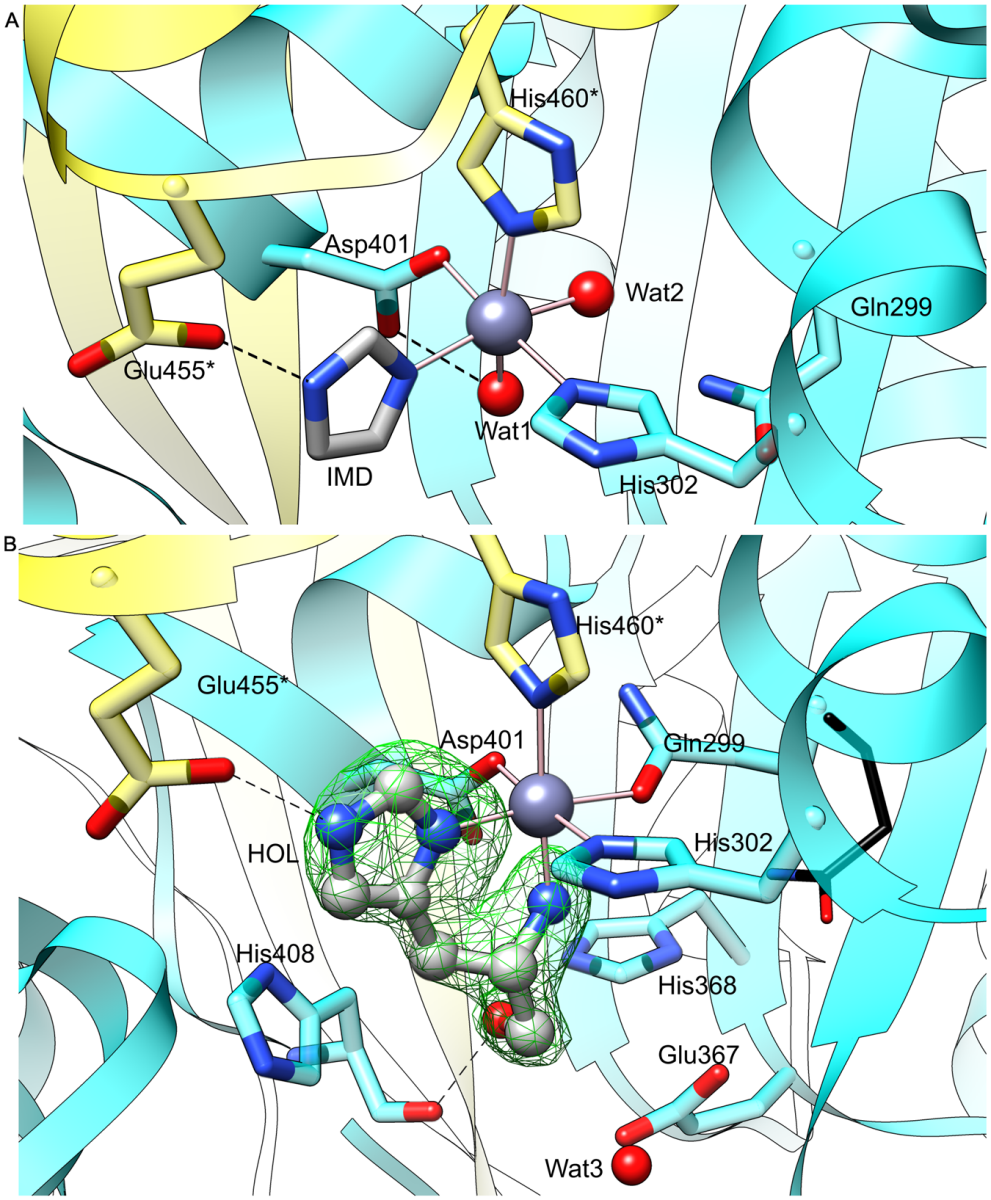
The active site of *Mt*HDH. Chain A is coloured cyan whereas chain B is yellow. Zn^2+^ coordination and imidazole (IMD) binding is shown in the A panel. The panel B depicts binding of the substrate, histidinol (HOL, ball-and-stick, green mesh is the omit *F*_*o*_ − *F*_*c*_ electron density map, contoured at 7σ level). Gln299 from superposed *Mt*HDH/IMD on *Mt*HDH/HOL complex is shown as thin black sticks in the panel B for comparison.

Each monomer of *Mt*HDH binds a single Zn^2+^ cation in a deep pocket between domains I, II, and IV* (asterisk indicates an element of the other protein subunit of the dimer), which is the active site of HDH enzymes^16^ (Fig. 4). In the structure with IMD, Zn^2+^ is octahedrally coordinated by two water molecules (Wat1 and Wat2), Nε of His302, Oδ of Asp401, Nε of His460*, and N of IMD.

IMD has been known as a weak, competitive inhibitor of HisD from *S. typhimurium*, with *K*_*i*_ = 1.5 mM^14^. In the *Mt*HDH complex, IMD creates two polar interactions, one with Zn^2+^ and one with Glu455*, which bind indirectly to domain II and directly to domain IV* (Fig. 4). IMD faces domain I but does not interact with it. It is possible that, for stability of the dimer, there must be a link between Zn^2+^ and Glu455*, as we failed to obtain a structure without IMD (or an IMD-derivative) added to the crystallisation solution.

### Binding of L-Histidinol drives structural rearrangements

HOL coordinates Zn^2+^ with N and Nδ atoms. More precisely, the N atom occupies the same position as Wat1 in the *Mt*HDH/IMD complex, whereas the imidazole moiety of HOL is bound in a manner similar to IMD (Fig. 4B). Like IMD, HOL also forms an H-bond with Glu455*. The O atom of HOL interacts with carbonyl O of His408 and Nε of His368.

It has been noted that the crystals were grown in acidic conditions (pH 5.2) in which the imidazole ring should be predominantly double-protonated. Based on the crystallisation screening, acidic conditions are necessary for *Mt*HDH to pack into a crystal lattice. This would greatly disfavour HOL binding. However, we observed that the best crystals were grown when *Mt*HDH was incubated with the ligands at pH 7.5 for 24–48 hours before the crystallisation was set up, which permits the single-protonated HOL to bind to the Zn^2+^-occupied site before crystallisation.

**Figure 5:**
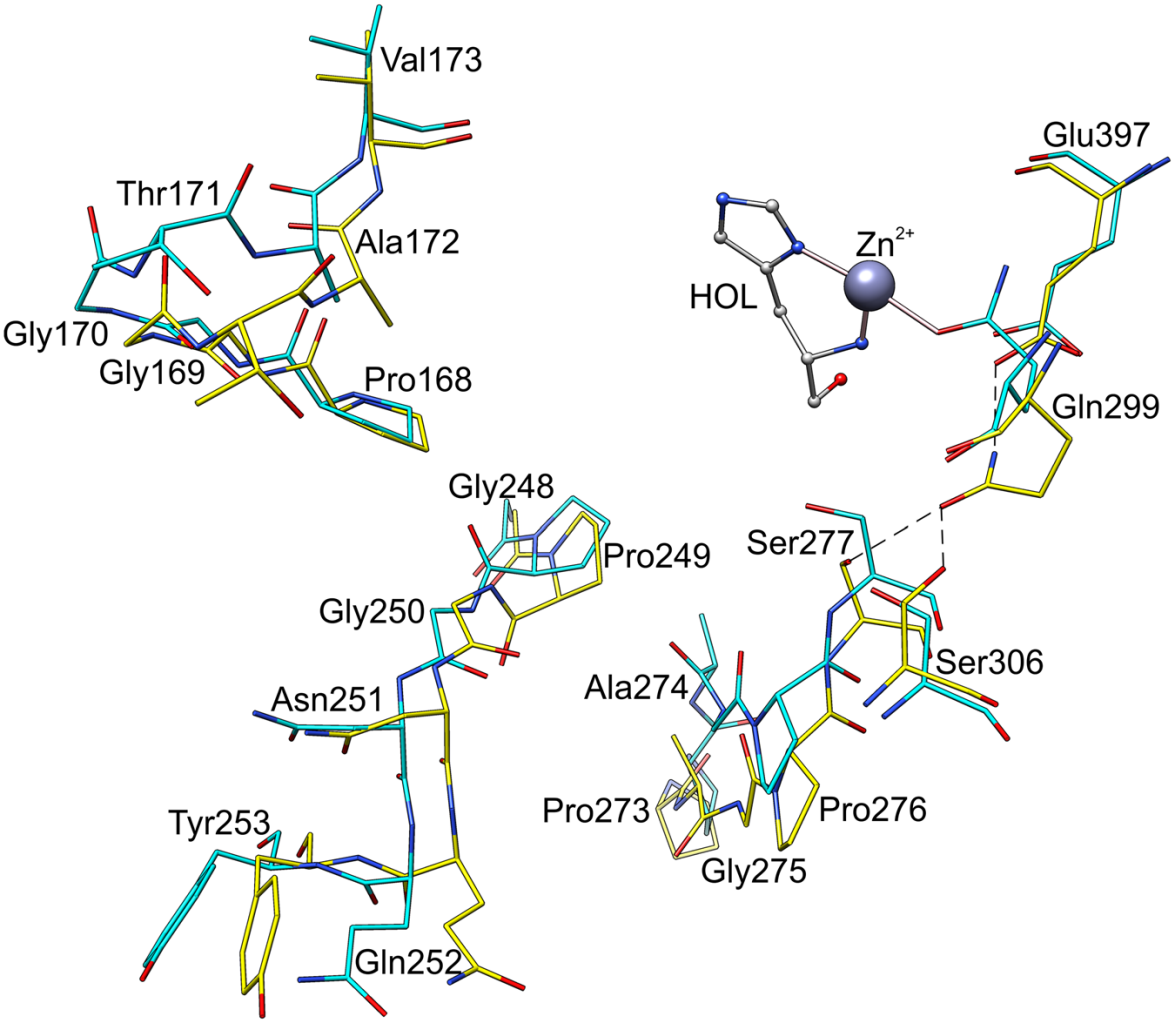
Fragments of the *Mt*HDH structure that undergo the most significant rearrangements upon HOL binding. Carbon atoms of the *Mt*HDH/IMD complex are yellow, whereas those of *Mt*HDH/HOL are in cyan. Zn^2+^ and HOL are added for reference.

Upon HOL binding, a significant change takes place in the coordination sphere of Zn^2+^. The Oε of Gln299 substitutes Wat2, meaning that, in the HOL complex, the metal is coordinated only by the protein and the substrate atoms (Figs 4 and 5). It has been known that NAD^+^ does not effectively bind to HDH enzymes in the absence of HOL, but the structural reason for that remained elusive. Based on the *Mt*HDH structures reported herein, we now have a better understanding of that feature. A close examination of the aligned structures allowed us to detect several differences (Fig. 5). One important change is a different conformation of Gln299. In the *Mt*HDH/IMD complex, Gln299 Nε interacted with the carboxylic O of Glu397, whereas the Oε was H-bonded to the Oγ of Ser277 and the Oγ of Ser306. In the complex with HOL, where Gln299 no longer binds Ser277, the latter has an altered conformation. It appears that Gln299 and Ser277 are the key players, because Ser277 takes part in NAD^+^ binding (see below). Although Gln299 belongs to domain II, this domain shows few conformational changes, whereas the majority of the rearrangements concern domain I. The most prominent movement involves a loop region from Pro273 to Ser277 (Fig. 5). A corresponding fragment was briefly mentioned by Barbosa *et al*.^16^. Other conformational changes involve loop Pro168-Val173 and the loop with the N-terminal side of helix α10, Gly248-Tyr253. Rearrangement of the latter region flips the ψ angle of Pro249 by almost 155°, from −53.8° in the *Mt*HDH/IMD complex to 151.3° in HOL complex. This feature has not been observed in *Ec*HisD^16^. The three mostly loop regions are very conserved among HDH enzymes (Fig. 2) and, as presented below, contribute to NAD^+^ binding.

### The structure of *Mt*HDH shows NAD^+^ binding that allows for a hydride transfer

To visualise the cofactor binding site, *Mt*HDH was cocrystallised after incubation with the product (His) to mimic the substrate (HOL) and NAD^+^ at pH 7.5 to allow for binding. If HOL was added instead of His, the increase in absorbance at 340 nm related to the production of NADH (not shown) confirmed that the enzymatic reaction had taken place. In the *Mt*HDH/His/NAD^+^ complex, His binds in the same manner as HOL, with the HOL and His O atoms lying at corresponding positions and creating similar contacts with His368 and carbonyl O of His408. Gln299 coordinates Zn^2+^ via Oε as it does in the complex with HOL.

**Figure 6:**
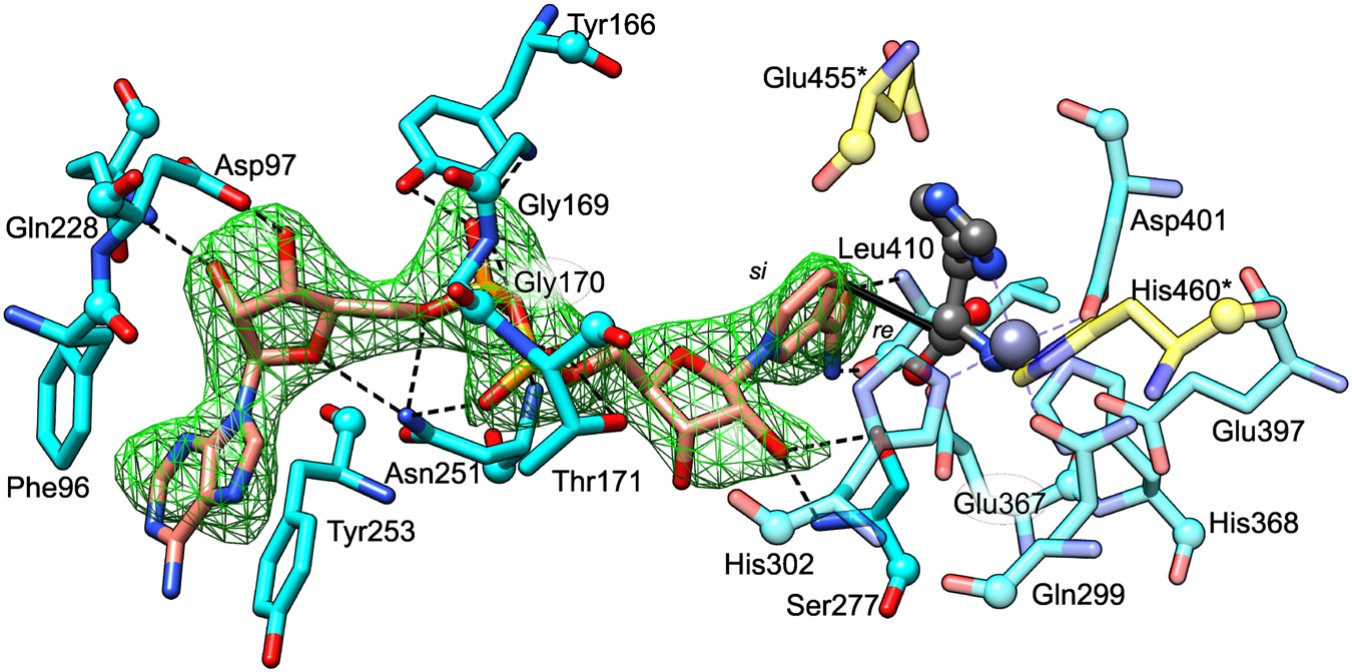
NAD^+^ binding by *Mt*HDH. NAD^+^ (salmon) is contoured with the *F*_*o*_ *− F*_*c*_ omit map (green mesh) at 4 σ level. The product, His (gray C atoms, ball-and-stick), mimics the position of HOL. Hydrogen bonds between the protein and NAD^+^ atoms are shown as dashed black lines, whereas the solid black line represents the path of hydride transfer. Residues from domain II are semitransparent, whereas those from domain IV* are yellow. Italicised *re* and *si* indicate the two prochiral faces of the C4 atom of nicotinamide.

In all six subunits in the asymmetric unit, a clear electron density—which agrees with the structure of NAD^+^—was found in clefts within domain I that lead to the His binding site (Fig. 6). The NAD^+^ adenine moiety is stacked between Phe96 (inter-ring distance ≈ 3.5 Å) and Tyr253 (≈ 3.4 Å). Going towards the His-binding site, there are 15 polar interactions between *Mt*HDH and NAD^+^. The adenosine ribose O2′ interacts with Nε of Gln288 and O3′ with Oδ of Asp97, whereas O4′ and O5′ both interact with Nδ of Asn251. The NAD^+^ pyrophosphate binds to Oη of Tyr166, Oγ of Thr171, and Nδ of Asn251, in addition to the backbone amides of Gly169, Gly170, Thr171, and Asn251. The nicotinamide-adjacent ribose, by its O2′, binds to Oγ and the amide of Ser277 (mentioned previously). Nicotinamide moiety binds to Oε of Glu 367 and Leu410, which belong to domain II. More specifically, the nicotinamide NH_2_ group interacts with carbonyl O and nicotinamide O with the backbone amide of Leu410. It is important to note that most of the residues that bind NAD^+^ (Gly169, Gly170, Thr171, Asn251, Ryr253, and Ser277) are within the loops pinpointed in the previous section as the fragments undergoing the most significant rearrangements upon HOL binding that “prepare” the enzyme for interaction with NAD^+^. In other words, thanks to the *Mt*HDH complexes, we know which conformational changes triggered by HOL binding enable the enzyme to bind NAD^+^.

The distance between the C4 atom of nicotinamide and the C of His is approximately 3.1 Å. Such proximity would allow for a hydride transfer from the C atom of HOL to the C4 of NAD^+^ and, subsequently, from HAL to another NAD^+^ molecule. As mentioned in the Introduction, the corresponding distance in the only other NAD^+^-containing HDH structure, *Ec*HisD [PDB ID: 1kae, ref. 16], was 4.9 Å, which resulted in the presence of unreacted HOL in the active site. In that structure, there are also far fewer binding interactions between the enzyme and NAD^+^. Only six direct and two water-mediated H-bonds anchor the cofactor to *Ec*HisD. Among them, conserved between *Ec*HisD and *Mt*HDH (corresponding residue parenthesised), are the following interactions: O2′ of adenosine ribose and Gln188 (Gln228); O4′ and O5′ of the same ribose with Nδ of Asn211 (Asn251); pyrophosphate with backbone N, Nδ of Asn211 (Asn251), and Oη of Tyr130 (Tyr166). Neither nicotinamide nor nicotinamide ribose interacted with the protein atoms in *Ec*HisD. This means that our structures of *Mt*HDH complement complexes of *Ec*HisD by showing the location and conformation of NAD^+^ that is very likely to reflect the reactive state.

Because NAD^+^ was located far from the reactive carbon of HOL in the *Ec*HisD complex and the nicotinamide ring was oriented nearly perpendicular to the C–Cα bond of HOL, it was difficult to determine on which face the hydride(s) might be accepted. Based on the *Mt*HDH structures, it is possible to unambiguously determine that the hydride is abstracted by the *re* face of nicotinamide C4 (Fig. 6).

**Figure 7:**
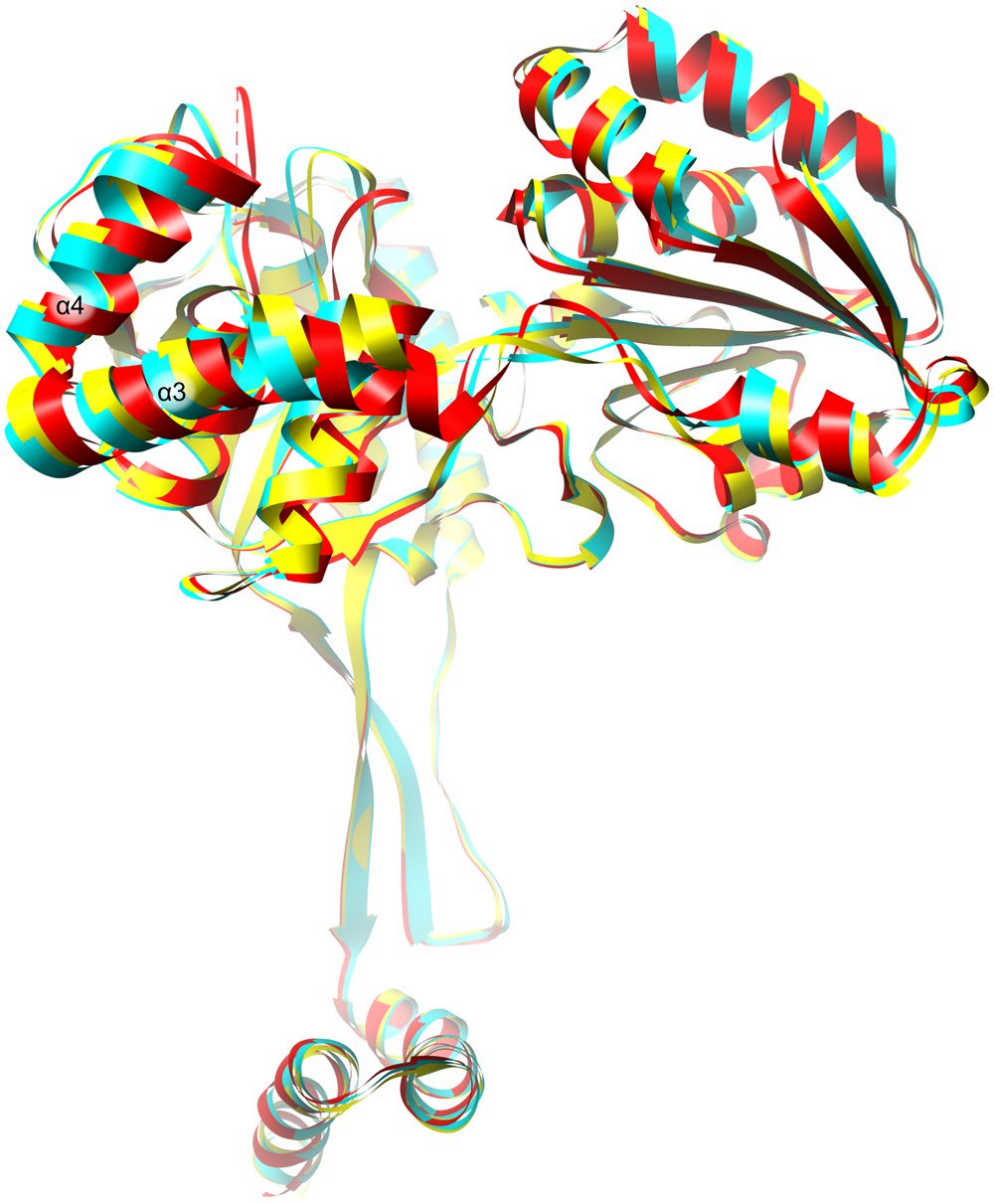
Superposition of the three states of *Mt*HDH: *Mt*HDH/IMD complex, yellow; *Mt*HDH/HOL, cyan; *Mt*HDH/His/NAD^+^, red.

The root-mean-square-deviation (rmsd) between the *Mt*HDH/HOL and *Mt*HDH/His/NAD^+^ complexes is 1.1 Å (419 Cα pairs within 3 Å distance), which would indicate more profound differences than between the *Mt*HDH/IMD and *Mt*HDH/HOL complexes (0.64 Å). However, as shown in the previous section, HOL binding involved crucial conformational changes, such as mainchain flips, whereas the subsequent binding of NAD^+^ only causes shifts of region, particularly the V-shaped pair of helices α3 and α4 (Fig. 7). The lock-and-key analogy postulated by Emil Fischer in 1894 and commonly used for enzymes can be modified for *Mt*HDH to lock-key-and-door. HOL binding unlocks the door and turns the knob (detailed but, *nomen est omen*, key changes) and allows NAD^+^ to bind, which “opens the door.” However, in the case of *Mt*HDH, “opening” does not mean that there are hinges between domains because the relative positions of the four domains remain unchanged throughout the course of the reaction.

### Plant versus bacterial HDHs

Biochemical studies of cabbage enzyme (*Bo*HDH) that is nearly identical to the *Mt*HDH showed that, functionally, plant HDHs behave similarly to the bacterial orthologs^30, 31, 38^. With the knowledge of the 3-D structures of *Mt*HDH, it is possible to adopt and update the mechanism proposed previously^12, 16, 39^ (Fig. 1). Namely, a proton is withdrawn from the HOL O atom by Nε of His368 (Base 1) that becomes double-protonated (it must also be protonated at Nδ, as it forms an H-bond with the carbonyl O of Glu278), and one hydride is abstracted by the first NAD^+^ molecule. The hybridisation of reactive carbon changes from sp^3^ to sp^2^, and HAL is formed. The “used” NADH dissociates and is replaced by the second NAD^+^ molecule. A water molecule (Wat3 in Fig. 4) is activated by Glu367 (Base 2) and performs a nucleophilic attack on the reactive carbon, forming a new C–O bond. Simultaneously, the HAL oxygen withdraws the proton back from Nε of His368 (H:Base 3, now acting as an H-donating acid), resulting in the formation of a *gem*-diol HAL hydrate with sp^3^-hybridised carbon. In the next step, His368 (Base 4) abstracts a proton from one of the hydroxyl groups of HAL hydrate, whereas the second NAD^+^ removes hydride from the reactive carbon, changing its hybridisation to sp^2^ and producing His.

Activation of HDH enzymes by Mn^2+^, reported for bacterial orthologues^15^, is very unlikely to be physiologically relevant in plants. Only a 10% increase of the *Salmonella typhimurium* enzyme activity was observed in 50 µM Mn^2+^, and a 50% increase was observed in 500 µM Mn^2+^. Such a high concentration corresponds to Mn-toxic acid soils, and while chloroplasts are second to vacuoles among organelles that accumulate Mn^2+^, a 10 µM concentration is already considered high^40^.

**Figure 8:**
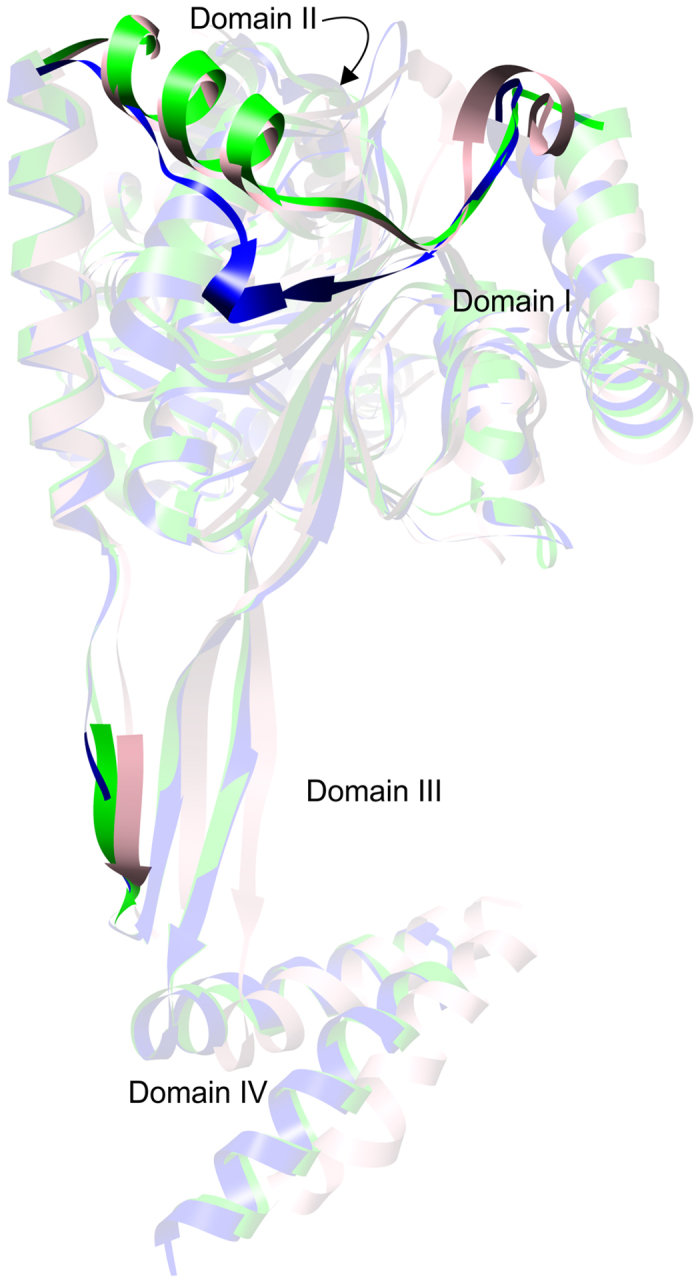
Structural comparison of the three examples of HDHs with a known three-dimensional structure. *Mt*HDH, blue; *Ec*HisD, green; and *Bs*HisD, pink are presented. Regions showing higher variability are in full colours, whereas those that align well are semitransparent.

Structurally, *Mt*HDH is similar to the two bacterial HDHs, *Ec*HisD and *Bs*HisD. There are, however, a few differences, mainly within domains I and III, as revealed by the superpositions of the *Mt*HDH/IMD complex with *Ec*HisD (PDB ID: 1k75) and *Bs*HisD (4g07). They are most prominent between residues 98–117 in *Mt*HDH and the corresponding residues 58–81 in *Ec*HisD and *Bs*HisD (Fig. 8). The two bacterial HDHs lack the β2 strand (in *Mt*HDH topology) and the short η5 helix that *Mt*HDH has. Furthermore, the subsequent residues 111–115 form a loop region in *Mt*HDH, whereas the corresponding fragment of prokaryotic HDHs is longer by two amino acids (Fig. 2) and forms a helix. Another significant difference is the presence of a two-stranded β-sheet within the domain III in *Mt*HDH, as opposed to the three-stranded β-sheet in *Ec*HisD and *Bs*HisD. In *Mt*HDH, only Val145 interacts as in a β-sheet. However, in all three enzymes, domain III extends the β-sheet of domain II (see above). It is also very interesting to note that the correlation of the sequence alignment (Fig. 2) with a structural comparison shows that HDHs often share a more significant similarity within the loop regions than within fragments of a more compact secondary structure.

Most plant chloroplast dehydrogenases prefer NADP(H) over NAD(H) as the dinucleotide cofactor. HDHs are unusual from that perspective. Cabbage *Bo*HDH, for instance, displays a 870-fold preference for NAD^+^ over NADP^+^^41^. The *Mt*HDH/His/NAD^+^ structure explains this preference. A formation of the *Mt*HDH/NADP^+^ complex is highly disfavoured because of severe steric hindrances between the O2′ phosphate and Gln228 (Fig. 6), in addition to the negative–negative charge repulsion.

HDH inhibitors have the potential to be antibacterial agents^6–9^. It will be exciting to see if similar compounds act against plant enzymes, too. Undoubtedly, further studies are required to examine the possibilities. In such light, this work, which updates and rectifies previous observations, particularly those related to the cofactor binding, is important because it may serve as a new scaffold for structure-based drug design. Now, thanks to the *Mt*HDH structures, the design may also include transition-state analogues, which are generally considered to be very powerful enzyme inhibitors^42, 43^.

## Methods

### Cloning, overexpression, and purification of *Mt*HDH

The total RNA was isolated from *M. truncatula* roots using the RNeasy Plant Mini Kit (Qiagen), and the transcriptome was transcribed into the coding DNA (cDNA) with SuperScript II reverse transcriptase (Life Technologies) using oligo dT (15 and 18) primers. The cDNA suited as a template for amplification of the sequence coding for *Mt*HDH ORF without the predicted N-terminal signal peptide (35 amino acids) by polymerase chain reaction (PCR). The signal peptide was recognised using the TargetP 1.1 server^44, 45^. The primers used (Forward: TACTTCCAATCCAATGCCTCCATTTCCATGGCAAATCCAATCAAAAC, Reverse: TTATCCACTTCCAATGTTATCATCTTGAAACCTGTCTGGCTTCTATG) allowed us to incorporate the insert into the pMCSG68 vector (Midwest Center for Structural Genomics) using a ligase-independent cloning method^46^. The pMCSG68 vector introduces a His_6_-tag, followed by the Tobacco Etch Virus (TEV) protease cleavage site and the Ser-Asn-Ala linker, which precedes the N-terminus of the expressed protein. The correctness of the insert was confirmed by DNA sequencing.

Overexpression was carried out in LB media supplemented with 150 μg/mL ampicillin in BL21 Gold *E. coli* cells (Agilent Technologies). The bacteria were cultured with shaking at 210 rpm at 37 °C until the OD_600_ reached 1.0. Then, the cultures were cooled down to 18 °C, and *Mt*HDH overexpression was induced by the addition of isopropyl-D-thiogalactopyranoside at a final concentration of 0.5 mM, which continued for 18 h. The cell pellet from the 2 L culture was centrifuged at 3,500 x g for 20 min at 4 °C and resuspended in 35 mL of binding buffer [50 mM Hepes-NaOH pH 7.5; 500 mM NaCl; 20 mM IMD; 1 mM tris(2-carboxyethyl)phosphine (TCEP)] and stored at −80 °C. The samples were thawed and the cells were disrupted by sonication (4 min of probe working time), using bursts of 4 s and 26 s intervals for cooling in an ice/water bath. The cell debris was pelleted by centrifugation at 25,000 x g for 40 min at 4 °C. The supernatant was applied to a 50 ml column packed with 4 mL of HisTrap HP resin (GE Healthcare) and plugged into VacMan (Promega), with a vacuum pump setup to accelerate the process. The resin-bound *Mt*HDH was washed five times with 40 mL of the binding buffer. The His_6_-tagged protein was eluted with 20 mL of elution buffer (50 mM Hepes-NaOH pH 7.5; 500 mM NaCl; 400 mM IMD; 1 mM TCEP). The His_6_-tag was cleaved with TEV protease (final concentration 0.1 mg/mL), and the IMD concentration was lowered to 20 mM by simultaneous dialysis overnight at 4 °C. The solution was applied again to HisTrap HP resin to remove the cleaved His_6_-tag and the His_6_-tagged TEV protease. The flow-through was collected, concentrated to 2.4 mL, applied on a HiLoad Superdex 200 16/60 column (GE Healthcare), and equilibrated with a buffer composed of 25 mM Hepes-NaOH pH 7.5, 100 mM KCl, 50 mM NaCl, and 1 mM TCEP.

### Crystallisation and diffraction data collection

A homogenous, dimeric fraction of *Mt*HDH was concentrated using Amicon concentrators (Millipore) to 8 mg/mL (based on A_280_ with the extinction coefficient of 37,400). Screening for crystallisation conditions was performed using a robotic sitting drop vapor diffusion (Mosquito). The most promising hits were optimized manually in hanging drops. For complexes with HOL and His/NAD, the ligands (20 mM final concentration) were added as buffered solutions (in 100 mM Hepes pH 7.5) to the protein sample and incubated overnight. Compositions of the reservoir solution and the volumes of protein:solution (in µl, parenthesised) were as follows: (i) *Mt*HDH/IMD: H1 mix from Morpheus Screen [Molecular Dimensions^47^], buffered by IMD/2-(N-morpholino)ethanesulfonic acid (MES) (4:2); (ii) *Mt*HDH/HOL: 100 mM MES, pH 5.2, 200 mM NaCl, 12% polyethylene glycol (PEG) 3350 (2:2); (iii) *Mt*HDH/His/NAD^+^: 100 mM MES, pH 5.2, 200 mM NaCl, 10% PEG 3350 (2:2). For the latter two complexes, cryoprotection was obtained by rapid soaking in crystallisation solution supplemented with 20% glycerol, unlike for the *Mt*HDH/IMD crystals that already grew in a cryoprotected solution. Flash-frozen crystals were stored in liquid nitrogen for diffraction data collection. Data were collected at 22-ID and 19-ID beamlines at the Advanced Photon Source, Argonne, USA. The diffraction images were processed with *XDS* ^48^. The statistics of the data collection and processing are summarized in Table 1. The presence on Zn^2+^ in the active site was confirmed by X-ray fluorescence scan (not shown). It is noteworthy that Zn^2+^ was not added during the enzyme preparation or to the crystallisation mixture, as such supplementation resulted in protein precipitation. Instead, it needed to be incorporated into the protein active site during protein overexpression and remained bound throughout the purification procedure. Because atomic displacement parameters of the Zn^2+^ cations refined to similar values as those of the neighbouring atoms, the metals were modelled at full occupancy in all three structures of *Mt*HDH. Moreover, efforts to obtain a structure with NAD^+^ alone were ineffective, which is consistent with previous reports stating that NAD^+^ binding occurs only to HOL-, HAL-, or His-bound enzymes^11, 12^.

**Table 1:** Data collection and refinement statistics.

*Mt*HDH	Zn^2+^, IMD	Zn^2+^, HOL	Zn^2+^, His, NAD^+^
**Data collection**			
Wavelength (Å)	1.0000	0.9763	1.0000
Space group	*P*2_1_	*P*2_1_	*P*2_1_
Unit cell parameters			
*a, b, c* (Å)	105.8, 142.8, 105.4	102.9, 139.2, 102.7	103.7, 139.1, 103.6
α, β, γ (°)	90, 120.2, 90	90, 119.2, 90	90, 119.5, 90
Resolution (Å)	40–2.25 (2.39–2.25)	50–1.97 (2.09–1.97)	50–2.59 (2.75–2.59)
Reflections collected/unique	622093/120737	1080052/175983	219001/76791
Completeness (%)	94.7 (73.8)	99.4 (97.4)	96.4 (90.9)
Multiplicity	5.2 (3.5)	6.1 (5.7)	2.9 (2.7)
*R*_meas_^a^(%)	8.6 (71.5)	8.5 (99.6)	10.7 (64.8)
< *I*/σ(*I)*>	15.7 (2.1)	13.7 (1.9)	10.3 (2.0)
**Refinement**			
*R*_free_ reflections	1208	1232	1152
No. of atoms (non-H)			
protein	19734	19694	19668
ligands	78	66	425
solvent	656	625	149
*R*_work_/*R*_free_ (%)	18.1/23.4	17.9/22.9	22.1/26.4
Average B-factor (Å^2^)	42.0	47.0	57.0
RMSD from ideal geometry			
bond lengths (Å)	0.014	0.015	0.013
bond angles (°)	1.65	1.69	1.74
Ramachandran statistics (%)			
favored	96.8	96.9	97.3
allowed	3.2	3.1	2.7
outliers	0.0	0.0	0.0
PDB code	5vlb	5vlc	5vld

Values in parentheses correspond to the highest resolution shell.

^a^ *R*_meas_ = redundancy independent R-factor^60^.

### Determination and refinement of the crystal structures

The crystal structure of *Mt*HDH was solved by molecular replacement with *Balbes* ^49^, using 2.7 Å resolution data (not reported here). *Phenix AutoBuild* ^50^ was used to build the initial model, which afterwards was placed inside the unit cell with the *ACHESYM* server^51^. *COOT* ^52^ was used for manual fitting in the electron density maps between rounds of model refinement in *Refmac* ^53^. A single *TLS* ^54, 55^ group was added to each chain in every structure, whereas non-crystallographic symmetry restraints were added only for the lowest-resolution *Mt*HDH/His/NAD^+^ complex. The possibility of crystal twinning was taken into account because the a- and c-unit cell dimensions are similar, and the β angle is very close to 120° (Table 1). Twinning was, however, excluded during the structure refinement in *Refmac* ^53^. Models were validated in *MolProbity* ^56^. The refinement statistics are listed in Table 1.

### Other software used

Molecular illustrations were created with UCSF *Chimera* ^57^, which also served for calculations of rmsds. Sequence alignment was performed in ClustalW^58^, whereas identities/similarities were calculated in BLAST^59^.
